# Personality and Personality Disorders in Urban and Rural Africa: Results from a Field Trial in Burkina Faso

**DOI:** 10.3389/fpsyg.2013.00079

**Published:** 2013-03-11

**Authors:** Jérôme Rossier, Abdoulaye Ouedraogo, Donatien Dahourou, Sabrina Verardi, Franz Meyer de Stadelhofen

**Affiliations:** ^1^University of LausanneLausanne, Switzerland; ^2^University of OuagadougouOuagadougou, Burkina Faso

**Keywords:** personality, personality disorders, five-factor model, cross-cultural psychology, cultural psychiatry

## Abstract

When conducting research in different cultural settings, assessing measurement equivalence is of prime importance to determine if constructs and scores can be compared across groups. Structural equivalence implies that constructs have the same meaning across groups, metric equivalence implies that the metric of the scales remains stable across groups, and full scale or scalar equivalence implies that the origin of the scales is the same across groups. Several studies have observed that the structure underlying both normal personality and personality disorders (PDs) is stable across cultures. Most of this cross-cultural research was conducted in Western and Asian cultures. In Africa, the few studies were conducted with well-educated participants using French or English instruments. No research was conducted in Africa with less privileged or preliterate samples. The aim of this research was to study the structure and expression of normal and abnormal personality in an urban and a rural sample in Burkina Faso. The sample included 1,750 participants, with a sub-sample from the urban area of Ouagadougou (*n* = 1,249) and another sub-sample from a rural village, Soumiaga (*n* = 501). Most participants answered an interview consisting of a Mooré language adaptation of the Revised NEO Personality Inventory and of the International Personality Disorders Examination. Mooré is the language of the Mossi ethnic group, and the most frequently spoken local language in Burkina Faso. A sub-sample completed the same self-report instruments in French. Demographic variables only had a small impact on normal and abnormal personality traits mean levels. The structure underlying normal personality was unstable across regions and languages, illustrating that translating a complex psychological inventory into a native African language is a very difficult task. The structure underlying abnormal personality and the metric of PDs scales were stable across regions. As scalar equivalence was not reached, mean differences cannot be interpreted. Nevertheless, these differences could be due to an exaggerated expression of abnormal traits valued in the two cultural settings. Our results suggest that studies using a different methodology should be conducted to understand what is considered, in different cultures, as deviating from the expectations of the individual’s culture, and as a significant impairment in self and interpersonal functioning, as defined by the DSM-5.

## Introduction

Several studies have observed that the structure underlying both normal personality (McCrae et al., 2005) and personality disorders (PDs) is stable across cultures (Dahourou and Rossier, 2008). A study conduced by Rossier et al. (2008) in nine African countries and Switzerland observed that the relationship between PDs and normal personality was stable across countries, thus suggesting that a dimensional approach based on normal personality traits may be useful for describing PDs in various cultural settings. PDs may be considered as an extreme trait level of a normal personality dimension, or as dysfunction associated with general personality traits (Rossier et al., 2005). In Africa, these studies were usually conducted with well-educated participants and using European language versions of the various measurement instruments. This meant that less privileged or preliterate samples were not usually taken into account. In order to study the relationship between personality and PDs in a more diverse sample from an urban and a rural area in Burkina Faso, the questionnaires assessing personality and PDs were adapted into Mooré, the language of the Mossi ethnic group, and the most frequently spoken local language in Burkina Faso, and administered as an interview.

This introduction will first present how the five-factor theory, of the structure and expression of personality traits, describes the relationship between personality traits and culture. This theory makes several assumptions concerning the impact of cultures on people’s personality structure and on how they express that personality in a specific environment. A second sub-section of this introduction will define PDs and how these disorders relate with normal personality traits. By definition, PDs are supposed to be culturally dependent and for this reason, a review of the very rare literature about the relationship between PDs – its underlying structure and expression – and culture will be provided.

### Personality traits and culture

Among personality models that describe personality traits, the five-factor model is certainly the most recognized and used among personality psychologists. This model postulates that five independent dimensions, called neuroticism, extraversion, openness, agreeableness, and conscientiousness, allow describing personality traits parsimoniously (Rossier et al., 2004). This model is founded on the five-factor theory, that postulates that personality domains are inherited dispositions and belong to people’s *basic tendencies*, but that the expression of these dispositions in terms of concrete manifestations is actualized in what McCrae and Costa (2008) called *characteristic adaptations*. These adaptations allow concrete behaviors to be expressed in a specific situation. These adaptations also allow this *personality system* to take into account external influences that moderate the expression of basic personality components. Expression of personality traits is a dynamic process that is the result of an interaction between the environment and basic tendencies. This interaction, however, does not have a direct impact on these basic tendencies and the model in itself seems quite robust across cultures. Indeed, the five basic personality dimensions were found in a large variety of cultures (McCrae et al., 2005). Several studies have indeed observed that the five-factor model of personality replicates across cultures and reaches structural equivalence, suggesting that the postulated dimensions are useful for describing personality in a large variety of cultures (Rössier, 2005). However, for comparing scores across cultures, scales have also to reach metric equivalence (same metric that implies that the intervals between units are of the same length across cultures) and full scale or scalar equivalence (same origin across cultures). Roughly, verifying that a scale reaches structural, metric, and scalar equivalence implies that all psychometric properties are similar across cultures. If only part of these characteristics are similar, mean scores cannot be compared across cultures because the absolute meaning of these scores remain unknown (Duarte and Rossier, 2008). A recent study conduced in nine French-speaking African countries and Switzerland on a sample of 4,278 participants suggested that the personality measurement used reached structural and metric equivalence but not scalar equivalence (Zecca et al., in press). This stability across cultures explains that correlations between these personality dimensions and several demographic variables, such as age or gender, are similar across cultures (McCrae et al., 2005).

The environment does not seem to have an impact on personality structures, but certainly affects the expression of personality traits, explaining the differences in mean personality profiles across cultures (Allik and McCrae, 2004). This regulation is possible through *characteristic adaptations* that allow people to express behaviors adjusted to their environment and to the expectations of their environment (for example, a rather hostile person can regulate his anger, activating emotional regulation resources, to comply with social norms that require peoples not being aggressive to each other). Difficulties in that regulation might explain maladjustment due to under- or over-expression of personality traits. This regulation also involves the self-concept as a representation of the self and of one’s interaction with one’s environment. Several authors have suggested that mean level differences across cultures could be partly due to different response styles (van Herk et al., 2004). Smith (2004) studied the relationship between values and acquiescence across cultures and observed that this response style explained a substantive amount of the variance. He suggested that this response style might be a stable cultural difference and that people from cultures commonly called collectivistic are also more prone to adopt an acquiescent style of response. In contradiction with this study, and with the study of Johnson et al. (2005) that suggested that the acquiescent response style might be associated with agreeableness and respect for hierarchy, the study of Verardi et al. (2010) has shown that acquiescence explains only a small part of the variance in social desirability in a sample of eight African countries and Switzerland. More recently Mõttus et al. (2012) tested whether the reference group effect might be a possible bias explaining mean score differences across cultures. The idea was that people have culture-specific standards and that they use these standards to rate themselves. This study conducted in more than 20 countries concluded that the reference group effect does not explain the mean self-rated differences. These studies suggest that differences across cultures are not simply due to differences in terms of response styles but might indeed be the result of a cultural adaptation.

### Personality disorders and culture

Personality disorders are usually conceived as personality systems or structures that are not well adapted to social requirements or function poorly in their specific environment (Magnavita, 2004). These difficulties are supposed to have an impact on several life domains (cognition, emotional and impulse regulation, and interpersonal functioning), to be stable over time, to induce a mental pain or have a negative impact on people’s well-being, and to have started during adolescence or early adulthood. In the DSM-IV-TR (American Psychiatric Association, 2000, p. 287) PDs are defined as “an enduring pattern of inner experience and behavior that deviates markedly from the expectations of the individual’s culture.” Ten PDs are described and grouped in three symptomatological clusters. Cluster A is characterized by odd and eccentric behaviors and includes paranoid, schizoid, and schizotypal PDs. Cluster B is characterized by dramatic and erratic emotions and behaviors and includes histrionic, narcissistic, antisocial, and borderline PDs. Finally, Cluster C is characterized by anxious and fearful feelings and attitudes and includes avoidant, dependent, and obsessive-compulsive PDs. The DSM Axis II model suffers from several weaknesses, such as excessive comorbidity, or poor convergent and discriminant validity (Clark, 2007). For example, using a screening questionnaire in a sample of 52 borderline outpatients, Verardi et al. (2008) observed that the mean number of PDs was 6.29, illustrating the important overlap across PDs. Several authors who have studied the underlying structure of PDs and associated symptoms questioned this grouping into clusters and suggested that PDs need to be redefined using a dimensional model (Rossier et al., 2008).

Concerning the underlying structure of PDs, Widiger et al. (1987) suggested considering three latent dimensions (or higher-order components), called social involvement, assertion vs. dominance, and anxious rumination. More recently, Blackburn et al. (2005) identified only two higher-order components, an anxious vs. inhibited dimension and an acting-out dimension. However, most studies suggest considering four higher-order components that Livesley et al. (1998) called emotional dysregulation, dissocial behavior, inhibition, and compulsivity. PDs can also be described using a dimensional model rooted in normal personality research (Rossier et al., 2008). Several studies investigated the relationship between the five-factor model and PDs and found meaningful associations (Widiger et al., 2002; Bagby et al., 2005). For example, patients suffering from a borderline PD usually score especially high on neuroticism and low on agreeableness and conscientiousness (Verardi et al., 2008). The provisional version of the DSM-5 proposes a hybrid dimensional-categorical model for PDs. The idea is to consider only six PD types, antisocial, avoidant, borderline, narcissistic, obsessive-compulsive, and schizotypal, adding for all patients (in particular for those who do not fit one of these types) a description in terms of pathological personality domains and traits. The five domains are negative affectivity, detachment, antagonism, disinhibition vs. compulsivity, and psychoticism, and include 25 maladaptive personality traits (Krueger et al., 2012). For all patients characterized by a specific personality type or by PD traits, it is crucial to consider the level of impairment in self and interpersonal functioning.

According to the DSM-IV-TR, PDs can be defined as an enduring and overwhelming pattern of inner experience, emotion, and behavior that is not in accordance with the expectations of the individual’s social and cultural environment. For this reason, culture could have an impact on the structure underlying PDs, reflecting the main difficulties associated with these disorders, and certainly having an impact on the expression of these disorders (Rossier et al., 2008; Rigozzi et al., 2009). Moreover, expectations of the social and cultural environment might vary quite drastically from one region to the other, and might induce a social judgment about this pattern of inner experience, emotion, and behavior. For all these reasons, prevalence rates might vary quite drastically across cultures. However, the cultural factors that impact these prevalence rates are unknown and remain to be discovered (Tseng, 2001). Concerning the structure underlying PDs across cultures, the structure postulated by Livesley et al. (1998) was replicated in several African, Asian, and European countries (Van Kampen, 2002; Zheng et al., 2002; Rigozzi et al., 2009). Moreover, Rossier et al. (2008) observed that correlations between PDs and the FFM domains and facets were very similar in nine African countries, China, and Switzerland.

According to Paris (1998), the prevalence rate of PDs from cluster C might be higher in collectivistic cultures due to an excessive social control likely to exacerbate the expression of avoidant, dependent, and obsessive-compulsive traits. On the other hand, PDs from cluster B might be higher in individualistic cultures due to a lack of social support and clear common values that might exacerbate emotional regulation difficulties and antisocial, histrionic, and narcissistic traits. Studies reporting data about PDs’ prevalence rates in non-Western countries are very scarce and concerned a limited number of cultures. Thuo et al. (2008) reported prevalence rates for PDs in psychiatric hospital patients globally lower in Kenya (20%) compared to the USA and Europe (between 45 and 66%). This was confirmed in a Portuguese sample of in-patients of African origin (Alexandre et al., 2010). Other studies suggested that the prevalence rates of some specific PDs, such as the antisocial PD, are lower in Asia (Tseng, 2001). However, these results might be due to a cultural impact on the method and criteria used. Indeed, Tang and Huang (1995) specified that there are important conceptual differences between Chinese and Western cultures regarding PDs. A recent study by Gupta and Mattoo (2012) conducted in an Indian sample of outpatients showed that the prevalence rates are globally lower than those reported for Western societies.

### Aims of the study

The aim of this study was to compare an urban and a rural sample of a non-Western society, and to study the impact of these two cultural settings on the structure and expression of normal personality, PDs, and on the relationship between normal and abnormal personality. The urban cultural setting is supposed to experience a process of rapid Westernization, and to promote more individualistic values and behaviors compared to the rural cultural setting more preserved from this Westernization. As an illustration of the relative cultural isolation of the rural area studied, electricity is not available there, and access to television is therefore very limited. However, many family members have moved to cities and frequently come back to their village. Comparing thus this urban and rural sample allows a study of the cultural transformation supposedly due to this Westernization. According to Paris’s (1998) hypotheses about the impact of culture on the prevalence rate of PDs, PDs of cluster C should be more frequent in the rural sample, and PDs of cluster B should be more frequent in the urban sample.

This study was conducted in Burkina Faso, a West African country south of the Sahara desert. There were 14,017,262 inhabitants in Burkina Faso in 2006 with a growth rate of 3.1%. The average life expectancy is 53.0. Mortality rates in Burkina Faso are among the highest in the world and the two main factors contributing to this high rate are poverty and weak health services. The Gross National Product was $483 per capita in 2007 and most of this income was concentrated in cities. A large proportion of the population was estimated to live below the poverty line and most of the population lives from agriculture. In Burkina Faso more then 60 different ethnic groups can be found. These ethnic groups form three large families: the Voltaïque family representing about 75% of the population, the Mandé family (about 15%), and the Sahélien family (about 10%). The most frequent religion is Islam (61% of the population), followed by Christianity (23%), and Animism (15%). According to Hofstede (2001), the West African region is characterized by high scores on power distance and low scores on individualism and long-term orientation. This culture might be described as accepting status differences, valuing the group over the individual, and valuing social obligations and traditions. Again, low individualism should be associated with low prevalence rate of PDs of cluster B but higher prevalence rate of PDs of cluster C (Paris, 1998). Burkina Faso was a former French colony, and French remains the official language. The French language is used systematically in school, in administration and to communicate with others from a different ethnic group.

Studying how personality and PDs might be assessed in an urban and rural Mossi cultural context seems especially interesting because there is no study in this area with less privileged or preliterate African samples. The assessment procedure had to be adapted and the assessment tools to be translated in a language that remains mainly an oral language. Assessing personality and PDs orally instead of using standardized questionnaire might have an impact on the assessment due to self-presentation strategies (for example, self-disclosure might more difficult during an interview). The assessment of the equivalence of the measurement used may give some indications about the impact of the cultural setting on these psychological characteristics and how cultural changes and language might affect how these characteristics might be assessed. This study might be of interest for Burknabé clinicians but also for all psychologists interested by the effects of globalization and culture on human personality characteristics.

## Materials and Methods

### Participants

The sample included 1,750 participants from Burkina Faso (834 women, 895 men, and 21 participants who did not indicate their gender), ranging from 17 to 95 years of age (*M* = 31.35; *SD* = 12.75). A large sub-sample came from the urban area of Ouagadougou (*n* = 1,249) and another sub-sample from a rural village, Soumiaga (*n* = 501), situated 170 km from Ouagadougou and about 15 km from Ouahigouya, the largest city of the northern part of Burkina Faso close to the border with Mali. It should be noted, that the gender distribution in the area of Ouagadougou was slightly different from that in Soumiaga. In Ouagadougou, women represented 41.1% of the participants whereas they accounted for 66.0% of the sample in Soumiaga, χ^2^(1) = 87.33, *p* < 0.001. This difference might be attributed the rural exodus which affects more men than women, and these proportions maybe considered as representative of the population of these two regions. Concerning the ethnic groups represented, 74.9% were Mossi (the largest ethnic group in Burkina Faso), 4.2% Bisa or Samo, 3.8% Gourounsi, 3.1% Bobo, 2.5% Senufo, 2.4% Dagari or Lobi, 2.3% Gurma, 0.9% Fulani or Peul, 0.5% Bwa, and 3.6% belonged to another ethnic group or did not indicate their ethnic group. About 52.5% of the participants were Muslims, 44.2% Christians, 1.1% Animists, and 1.4% were from another religion (it should be noted that part of the Muslims or Christians might also be Animists but do not declare it; monotheist religions are perceived as more socially desirable). Concerning the level of education, 487 had no education (or less than 6 years of school), 246 finished compulsory school, 332 finished high school, 99 finished an apprenticeship, and 471 were now at university or had obtained a university degree. The level of education of the participants in Ouagadougou who completed the French-version of the instruments was much higher, χ^2^(5) = 1005.39, *p* < 0.001. Concerning the participants who were interviewed in Mooré, those from the Ouagadougou area also had a higher level of education, χ^2^(4) = 277.28, *p* < 0.001. In fact, the proportion of participants without any education was especially high in Soumiaga (*n* = 340, 69.0%). In Soumiaga, young people, older people, and women were overrepresented. About 39.8% of the participants were married, 38.9% were not married, and 21.3% were in another familial situation or did not indicate this information. About 43.1% of the participants had children and 34.7 reported having no children (number of children: *M* = 3.25; *SD* = 3.44).

### Measures

#### NEO personality inventory revised

The NEO-PI-R (Costa and McCrae, 1992) is a self-rating questionnaire of 240 items measuring the five major personality dimensions or domains: neuroticism (N), extraversion (E), openness to experience (O), agreeableness (A), and conscientiousness (C). Each domain is made up of six facets. N includes anxiety (N1), hostility (N2), depression (N3), self-consciousness (N4), impulsiveness (N5), and vulnerability (N6). E includes warmth (E1), gregariousness (E2), assertiveness (E3), activity (E4), excitement seeking (E5), and positive emotions (E6). O includes fantasy (O1), esthetics (O2), feelings (O3), actions (O4), ideas (O5), and values (O6). A includes trust (A1), straightforwardness (A2), altruism (A3), compliance (A4), modesty (A5), and tender-mindedness (A6). C includes competence (C1), order (C2), dutifulness (C3), achievement (C4), self-discipline (C5), and deliberation (C6). “I have sometimes experienced a deep sense of guild or sinfulness” is an example of item of the depression (N3) facet scale. Responses are made on a five-point Likert scale, ranging from *strongly disagree* to *strongly agree*. We used the validated French language version of the NEO-PI-R (Rolland and Petot, 1998) or a Mooré translation of the NEO-PI-R done especially for this research with the agreement of the publisher and of the authors. The internal reliabilities reported by the validation study of the French-version of the NEO-FFI-R ranged from 0.84 to 0.92 for the five personality dimensions (*Mdn* = 0.88) and ranged from 0.54 to 0.83 for the facet scales (*Mdn* = 0.72; Rossier et al., 2004). In Burkina Faso the reliabilities reported for the domains (*Mdn* = 0.79) and for the facet scales (*Mdn* = 0.52) were slightly lower (Rossier et al., 2005).

#### International personality disorders examination screening questionnaire

The IPDE is an instrument developed by the WHO and tested in an international field trial involving four continents including Africa (Loranger et al., 1994; Loranger, 1999). The screening questionnaire is a self-rating instrument made up of 77 true/false items measuring the ten DSM-IV PDs: paranoid, schizoid, schizotypal, antisocial, borderline, histrionic, narcissistic, avoidant, dependant, and obsessive-compulsive. For example, “I often seek advice or reassurance about everyday decisions” is an item of the dependant PD scale. A score of 3 or above on any PD scale suggests the presence of a PD, usually investigated further with the associated interview. We used the validated French language version of the IPDE screening questionnaire (Loranger et al., 1997) or a translation into Mooré done especially for this study. For the French-version of the IPDE, the reliabilities are usually low due to the heterogeneity of PD symptomatology. In a large African sample, they range from 0.32 to 0.56 (*Mdn* = 0.45; Rossier et al., 2008).

### Translations

In order to translate the NEO-PI-R and the IPDE into Mooré, the authors of this study tried to follow Geisinger’s guidelines (1994) as closely as possible. A bilingual linguist, specialist in the Mooré tongue, made a first translation of the two instruments. The second author of this article, a native Mooré-speaking Burkinabé, corrected this translation. The Mooré version of both instruments was then pre-tested by the interviewers both in Ouagadougou and Soumiaga, under the supervision of the third author in Ouagadougou and under the supervision of the second author in Soumiaga. After these pre-tests, group sessions were conducted in both locations with the participation of the fourth and the fifth authors in order to adjust the translations, and to adapt them to some regional specificities (Mooré is mainly an oral language that varies slightly from one region to the other). These group sessions were also organized in order to ensure that all interviewers correctly understood each item. A Mooré-speaking English teacher was commissioned to produce a back-translation. This back-translation was only delivered 2 years later. The field trial of our financed 30 month project in Burkina Faso was already finished and this back-translation could unfortunately not be used.

### Procedure

A sub-sample completed only the French-version of the NEO-PI-R (*n* = 496). This sub-sample was recruited at the University of Ouagadougou, in a school for higher education, and among the relatives and neighbors of the psychology students of the Department of Psychology of the University of Ouagadougou. This sub-sample consisted of well-educated people who are fluent in French. Another sub-sample completed the French-version of both the NEO-PI-R and of the IPDE (*n* = 245). They were recruited by the master students in psychology of the University of Ouagadougou among their relatives and neighbors. These participants were again well-educated and fluent in French. For another sub-sample a Mooré version of both the NEO-PI-R and IPDE were used (*n* = 1,009). Participants from the Ouagadougou area (*n* = 508) were interviewed by master students in psychology. Participants from the Soumiaga region (*n* = 501) were interviewed by social workers, teachers, nurses, and adult trainers, hired in the larger region of Ouahigouya. The field trial in Ouagadougou was conducted under the supervision of the third author and the field trial in Soumiaga under the second author. The interviewers in both regions were trained by the local supervisor and the last two authors. This training included a presentation of the five-factor model of personality, of the PDs as described in the DSM-IV, and training on the use of the NEO-PI-R and IPDE. This training lasted between 12 and 18 h. The project was approved by the local state authorities and by the traditional village council for Soumiaga. This research complies with the ethical rules of the Swiss Society for Psychology (SSP).

## Results

The internal consistencies for the main personality domains for the validated French-version and for the experimental Mooré version of the NEO-PI-R were similar and ranged respectively from 0.69 to 0.84 (*Mdn* = 0.79) and from 0.72 to 0.85 (*Mdn* = 0.78). For the facet scales, internal consistencies were higher considering the French-version, from 0.22 to 0.66 (*Mdn* = 0.53), compared to the Mooré version, 0.25–0.75 (*Mdn* = 0.43). Comparing the internal consistencies of the Mooré version in an urban vs. rural area, these consistencies were slightly higher in the urban area for the domains, 0.62–0.79 (*Mdn* = 0.75), and facet scales, 0.08–0.57 (*Mdn* = 0.40), compared to the rural area of Soumiaga, where the consistencies ranged from 0.64 to 0.77 (*Mdn* = 0.68) for the domains, and from 0.15 to 0.62 (*Mdn* = 0.39) for facet scales. Skewness and kurtosis values for the French and the Mooré versions of the NEO-PI-R were all below 1 in absolute magnitude, except for the trust facet scale of the Mooré version which was associated with a slightly higher kurtosis (*K* = 1.06). Some differences were observed between the urban and rural areas using the Mooré version, with a kurtosis for extraversion in the urban area (*K* = 1.57), and a skewness (*S* = 1.24) and kurtosis (*K* = 2.21) for conscientiousness in the rural area above 1.

The internal consistencies of the 10 PD scales of the validated French and experimental Mooré versions of the IPDE were also similar and ranged respectively from 0.23 to 0.56 (*Mdn* = 0.44) and from 0.27 to 0.52 (*Mdn* = 0.39). Comparing the internal consistencies of the Mooré version in an urban vs. rural area, these consistencies were slightly higher in the urban area, 0.30–0.60 (*Mdn* = 0.45), compared to the rural area, 0.20–0.48 (*Mdn* = 0.27). Skewness and kurtosis values for the 10 PD scales of the French and Mooré versions of the IPDE were all below 1 in absolute magnitude, indicating that values tend to be normally distributed. No difference was observed between the urban and rural areas concerning distribution properties.

### Impact of demographic variables

To establish whether psychological constructs are stable across different contexts, one can study the stability of the relationships between these constructs and some demographic variables (Van de Vijver and Leung, 1997). For this reason, correlations with age and gender differences were studied in the sub-sample from Ouagadougou who answered in French and in the two sub-samples from Ouagadougou and Soumiaga who answered in Mooré. For the sub-sample who answered in French, we observed that age correlated with all personality dimensions (see Table 1). Concerning the correlations for the two sub-samples who answered in Mooré, there was almost no correlation between personality domains and age. Only one correlation was observed between age and extraversion in the urban sub-sample. Concerning correlations between age and PDs, significant correlations were observed only with the schizoid and schizotypal PDs in the rural sub-sample. Concerning the facet scales of the NEO-PI-R, correlations were all very low, with a mean correlation in absolute value below 0.10 for all three sub-samples. Interestingly the profiles of correlations for PDs and personality facet scales with age were similar for the two urban sub-samples (ρ = 0.60) but unrelated for the rural and the two urban sub-samples (ρ < |0.05|).

**Table 1:** **Partial correlations with age and means and standard deviations for women and men in the three sub-samples**.

Dimensions	Urban sub-sample assessed in French			Urban sub-sample assessed in Mooré			Rural sub-sample assessed in Mooré		
	(NEO-PI-R: *n* = 741; IPDE: *n* = 245)			(*n* = 508)			(*n* = 501)		
	*r*	Women	Men	*r*	Women	Men	*r*	Women	Men
Neuroticism	−0.15***	105.66 (16.14)	102.62 (16.22)	−0.04	99.38 (13.61)	94.41 (15.06)	−0.06	99.67 (11.49)	99.70 (10.32)
Extraversion	−0.09*	104.64 (15.06)	106.56 (14.90)	−0.10*	99.80 (11.90)	103.73 (11.91)	−0.08	121.29 (10.09)	119.87 (8.84)
Openness	−0.15***	108.77 (12.93)	109.18 (11.59)	−0.04	111.80 (14.63)	111.29 (14.01)	−0.08	134.99 (10.20)	132.90 (8.78)
Agreeableness	0.18***	118.02 (13.75)	116.12 (14.24)	0.05	115.66 (17.00)	113.21 (14.77)	−0.08	129.28 (8.6)	125.66 (9.87)
Conscientiousness	0.14***	114.85 (16.81)	115.30 (16.30)	0.05	108.66 (14.94)	110.90 (15.43)	−0.08	123.47 (11.68)	124.03 (10.11)
Paranoid	0.04	4.05 (1.26)	3.77 (1.55)	−0.04	3.40 (1.55)	3.35 (1.65)	0.04	2.98 (1.17)	3.34 (1.16)
Schizoid	0.03	2.99 (1.20)	2.63 (1.35)	0.00	2.74 (1.45)	2.39 (1.38)	0.13**	1.21 (1.03)	1.02 (0.96)
Schizotypal	−0.12	2.85 (1.70)	3.13 (1.76)	−0.06	3.27 (1.83)	3.13 (1.79)	0.12**	2.66 (1.29)	2.78 (1.38)
Antisocial	−0.09	1.79 (1.31)	1.82 (1.13)	−0.06	2.46 (1.56)	2.54 (1.53)	0.08	1.52 (1.16)	1.92 (1.42)
Borderline	−0.09	3.66 (1.85)	3.62 (2.07)	0.01	4.59 (2.09)	4.19 (2.10)	0.05	4.97 (1.66)	4.98 (1.70)
Histrionic	0.01	2.84 (1.60)	3.11 (1.63)	−0.08	3.57 (1.65)	3.66 (1.71)	0.06	2.79 (1.22)	2.89 (1.31)
Narcissistic	−0.09	4.94 (1.92)	5.05 (1.75)	−0.07	4.59 (2.02)	4.89 (2.03)	−0.05	5.15 (1.54)	5.58 (1.61)
Avoidant	−0.04	5.21 (1.47)	4.79 (1.78)	−0.02	4.99 (1.82)	4.60 (1.87)	−0.03	4.94 (1.37)	5.19 (1.39)
Dependent	−0.04	3.02 (1.61)	3.22 (1.67)	0.01	4.28 (1.77)	4.29 (1.75)	−0.04	5.78 (1.11)	5.11 (1.15)
Obsessive-compulsive	−0.02	3.64 (1.45)	3.59 (1.43)	−0.02	3.43 (1.58)	3.73 (1.68)	0.05	3.63 (1.19)	3.88 (1.28)

*p < 0.05. **p < 0.01. ***p < 0.001.

Concerning differences between women and men for personality domains after controlling for age, no non-negligible (η^2^ ≥ 0.01) and significant difference was observed for the French urban sub-sample. For the Mooré urban sub-sample women scored non-negligibly and significantly higher on neuroticism, *F*(1, 483) = 13.24, *p* < 0.001, η^2^ = 0.03, and lower on extraversion, *F*(1, 483) = 14.18, *p* < 0.001, η^2^ = 0.03. Finally, for the Mooré rural sub-sample women scored non-negligibly and significantly higher on agreeableness, *F*(1, 488) = 15.88, *p* < 0.001, η^2^ = 0.03. For facet scales only one difference was associated with at least a medium effect size (η^2^ ≥ 0.06), women scored non-negligibly and significantly lower on E5, *F*(1, 737) = 59.51, *p* < 0.001, η^2^ = 0.08, for the French urban sub-sample. For the remaining 89 comparisons, only 19 were associated with a small effect size. When comparing the mean profile of women across the three sub-samples, and comparing the mean profile of men across the three sub-samples, we observed high similarities (ρ ≥ 0.90). This similarity was much higher than the similarities between women or men in each sub-samples (ρ ranging from 0.42 to 0.84, *Mdn* = 53). Concerning PDs, for the paranoid PD a difference was observed only with the Mooré rural sub-sample, *F*(1, 482) = 10.31, *p* = 0.001, η^2^ = 0.02, with men scoring higher than women. For the schizoid PD, men scored slightly lower in all three sub-samples (η^2^ ranged from 0.01 to 0.02). For the antisocial PD, men scored higher only for the Mooré rural sub-sample, *F*(1, 482) = 9.77, *p* = 0.002, η^2^ = 0.02. For the borderline PD, women scored higher only in the Mooré urban sub-sample, *F*(1, 474) = 4.72, *p* = 0.03, η^2^ = 0.01. For the narcissistic PD, men scored higher only in the Mooré rural sub-sample, *F*(1, 480) = 9.44, *p* = 0.002, η^2^ = 0.02. For the avoidant PD, women scored slightly higher in both urban sub-samples (η^2^ ranged from 0.01 to 0.02). Finally, for the dependent PD, women scored much higher only in the Mooré rural sub-sample, *F*(1, 480) = 38.82, *p* < 0.001, η^2^ = 0.08.

The pattern of correlations with age did vary across regions. However, mean personality profiles of women and men differed but were similar across the three sub-samples. These correlations and differences were relatively small, and this has been previously attributed to the fact that “traditional Burkinabè society is a tribal society, in which the place of the individual is defined more in terms of social than of personal criteria” (Dahourou et al., 1995, p. 423). In such as context, a description in terms of personality traits might just be less relevant.

### The five-factor model structure across languages and regions

To determine whether the five-factor model of personality replicates across languages, we conducted a principal components exploratory factor analysis with varimax rotation on the 30 facets of the NEO-PI-R for the French and Mooré sub-samples. For the French sub-sample, Cattell’s scree test suggested five dimensions explaining 47.7% of the variance. The varimax rotation resulted in factors related to neuroticism, *r* = 0.94, extraversion, *r* = 0.90, openness, *r* = 0.76, agreeableness, *r* = 0.75, and conscientiousness, *r* = 0.82. For the Mooré sub-sample, Cattell’s scree test suggested three or four dimensions, with five dimensions explaining 55.1% of the variance. The varimax rotation resulted in factors related to neuroticism, *r* = 0.95 and conscientiousness, *r* = 0.81. Extraversion, *r* = 0.77, openness, *r* = 0.88, and agreeableness, *r* = 0.72, were all related to the same factor. To assess the cross-language replicability, the Mooré loading matrix was subjected to an orthogonal Procrustes rotation (McCrae et al., 1996) using the French matrix as the target. We found a total congruence coefficient of 0.78. The congruence coefficients for factors ranged from 0.58 to 0.86 (*Mdn* = 0.80) and from 0.34 to 0.98 (*Mdn* = 0.80) for facet scales. We also compared the structure found for the Mooré urban sub-sample with the structure found for the French urban sub-sample and observed a total congruence coefficient of 0.81. The congruence coefficients for factors ranged from 0.56 to 0.90 (*Mdn* = 0.82) and from 0.16 to 0.98 (*Mdn* = 0.85) for facet scales.

To determine if the five-factor model of personality replicates across regions, we conducted a principal components exploratory factor analysis with varimax rotation on the 30 facets of the NEO-PI-R for the Mooré urban and rural sub-samples. For the urban sub-sample, Cattell’s scree test suggested five dimensions explaining 50.7% of the variance. The varimax rotation resulted in factors related to openness, *r* = 0.80, conscientiousness, *r* = 0.83, and extraversion, *r* = 0.71. Neuroticism and agreeableness both loaded on the same factor, *r* = −0.55 and *r* = 0.56. For the rural sub-sample, Cattell’s scree test also suggested five dimensions, explaining 51.0% of the variance. The varimax rotation resulted in factors related to neuroticism, *r* = 0.84 and openness, *r* = 0.70. Extraversion, *r* = 0.58, agreeableness, *r* = 0.75, and conscientiousness, *r* = 0.86, were related to the same factor. To assess the cross-region replicability, the rural loading matrix was subjected to an orthogonal Procrustes rotation using the urban matrix as the target. We found a total congruence coefficient of 0.76. The congruence coefficients for factors ranged from 0.56 to 0.86 (*Mdn* = 0.76) and from 0.04 to 0.98 (*Mdn* = 0.79) for facet scales.

Previous analyses with the Mooré sub-samples have suggested that three personality domains might load on the same factor. For this reason, we analyzed whether a three-factor solution would be more stable across languages and regions. Globally, the structure was much more stable across languages with a total congruence coefficient of 0.91, than across regions with a total congruence coefficient of 0.78. This suggests that structural equivalence is not reached and that the meaning of personality dimensions and traits might be slightly different from one region to the other, and from one language to the other. If a psychometric measurement does not reach structural equivalence, it also does not reach metric or scalar invariance.

### PD underlying structure across languages and regions

To study the stability of the structure underlying PDs across the two languages and the two regions, a principal components exploratory factor analysis with varimax rotation on the 10 PDs was conducted for the French (*n* = 239) and the Mooré (*n* = 1,009) sub-samples. For the French sub-sample, Cattell’s scree test suggested three, four, or five dimensions. Considering previous studies conducted in African countries (Rigozzi et al., 2009), a four-factor solution was taken into consideration, which explained 64.4% of the variance. After a varimax rotation, the borderline, histrionic, and dependent PDs loaded on the first factor, the schizoid, schizotypal, and avoidant PDs loaded on the second factor, the narcissistic and obsessive-compulsive PDs loaded on the third factor, and the paranoid and antisocial PDs loaded on the last factor (see Table 2). For the Mooré sub-sample, Cattell’s scree test suggested three or four dimensions, with four dimensions explaining 66.9% of the variance. After a varimax rotation, borderline, narcissistic, and obsessive-compulsive PDs loaded on the first factor, avoidant and dependent PDs on the second factor, paranoid and schizotypal on the third factor, and schizoid, antisocial, and histrionic PDs loaded on the last factor. Applying a Procrustes rotation to the Mooré matrix, using the French matrix as the target, we observed a total congruence coefficient of 0.81. The congruence coefficients for factors ranged from 0.59 to 0.92 (*Mdn* = 0.88) and from 0.64 to 0.99 (*Mdn* = 0.82) for PDs.

**Table 2:** **Structure underlying PDs using a French-version of the IPDE screening questionnaire or a Mooré version of the IPDE administered through an interview, after Procrustes rotation, in the urban area of Ouagadougou**.

PDs	French-version of the IPDE				Mooré version of the IPDE				
	in Ouagadougou				in Ouagadougou			
	F1	F2	F3	F4	F1	F2	F3	F4	CCs
Paranoid	−0.28	0.37	**0.47**	**0.50**	−0.07	**0.62**	**0.55**	0.21	0.86
Schizoid	0.02	**0.83**	−0.11	0.00	−0.03	**0.51**	−**0.45**	**0.55**	0.64
Schizotypal	0.12	**0.69**	0.30	0.09	−0.22	**0.40**	0.32	**0.59**	0.64
Antisocial	0.24	0.05	0.02	**0.88**	0.22	0.29	0.19	**0.64**	0.92
Borderline	**0.56**	0.37	0.12	0.38	**0.51**	0.25	**0.48**	0.16	0.84
Histrionic	**0.74**	−0.07	0.16	0.23	**0.59**	0.05	−0.14	**0.61**	0.80
Narcissistic	**0.44**	−0.04	**0.58**	0.18	0.37	0.00	**0.65**	0.26	0.99
Avoidant	0.34	**0.51**	0.22	0.20	**0.49**	**0.60**	0.28	−0.28	0.81
Dependent	**0.74**	0.28	0.08	−0.10	**0.65**	0.14	0.32	−0.32	0.89
Compulsive	0.13	0.16	**0.82**	−0.03	**0.41**	−0.03	**0.52**	**0.41**	0.70
Congruence coefficients (CCs)					0.92	0.88	0.84	0.59	0.81

*Loadings equal or above 0.40 in absolute magnitude are in bold*.

In order to assess the stability of the structure underlying PDs across the two regions, a principal components exploratory factor analysis with varimax rotation on the 10 PDs was conducted for the urban (*n* = 508) and the rural (*n* = 501) sub-samples. For the urban sub-sample, Cattell’s scree test suggested three dimensions. However, four dimensions were considered which explained 71.1% of the variance. After a varimax rotation, the borderline, avoidant, and dependent PDs loaded on the first factor, the antisocial, borderline (secondary loading), histrionic, narcissistic, and obsessive-compulsive PDs loaded on the second factor, the paranoiac and schizotypal PDs on the third factor, and the schizoid PDs on the last factor (see Table 3). For the rural sub-sample, Cattell’s scree test suggested four dimensions which explained 59.9% of the variance. After a varimax rotation, the paranoid, antisocial, borderline, narcissistic, and obsessive-compulsive PDs loaded on the first factor, the schizoid and histrionic PDs on the second factor, the schizoid (secondary loading) and dependent PDs on the third factor, and a clear loading was observed for the last factor. Applying a Procrustes rotation to the matrix from Soumiaga, using the matrix from Ouagadougou as the target, a total congruence coefficient of 0.90 was observed. The congruence coefficients for factors ranged from 0.88 to 0.92 (*Mdn* = 0.90) and from 0.73 to 0.99 (*Mdn* = 0.93) for PDs. The congruence coefficients of only three PDs were below 0.90, and only one was below 0.80. Overall, the structure underlying PDs was stable across regions but not across languages. The meaning of the underlying dimensions of the experimental Mooré version of the IPDE seems similar in both regions.

**Table 3:** **Structure underlying PDs in an urban and a rural sample, after Procrustes rotation, in Burkina Faso using a Mooré version of the IPDE administered through an interview**.

PDs	Ouagadougou				Soumiaga				
	F1	F2	F3	F4	F1	F2	F3	F4	CCs
Paranoid	0.27	0.11	**0.87**	0.03	**0.48**	0.01	**0.63**	0.01	0.93
Schizoid	0.00	0.06	0.11	**0.96**	−0.05	0.07	−0.01	**0.88**	0.99
Schizotypal	−0.06	0.27	**0.65**	**0.48**	−0.08	0.16	**0.59**	0.40	0.99
Antisocial	0.12	**0.63**	**0.46**	0.10	0.07	**0.48**	**0.48**	0.22	0.97
Borderline	**0.51**	**0.50**	0.33	0.06	**0.52**	**0.48**	0.17	0.01	0.98
Histrionic	0.11	**0.84**	0.03	0.06	−0.05	**0.73**	−0.14	0.23	0.93
Narcissistic	**0.46**	**0.54**	0.26	−0.03	0.15	**0.45**	**0.50**	−0.24	0.81
Avoidant	**0.86**	0.00	0.19	−0.03	**0.50**	−0.06	**0.47**	−0.11	0.85
Dependent	**0.78**	0.36	−0.04	−0.04	**0.78**	−0.12	−0.39	0.24	0.73
Compulsive	**0.47**	**0.53**	0.16	0.20	18	**0.68**	0.22	−0.13	0.81
Congruence coefficients (CCs)					0.91	0.92	0.88	0.88	0.90

*Loadings equal or above 0.40 in absolute magnitude are in bold*.

### Metric and scalar equivalence of the IPDE across regions

Having established that the structure underlying the Mooré version of the IPDE was stable across the two regions, we further investigated the metric and scalar equivalence conducting a multi-group confirmatory factor analysis. All three levels of equivalence have to be achieved to conduct meaningful group comparisons (Rössier, 2005). The model took into account the ten PDs scales, four latent dimensions, and all loadings of the exploratory factor analysis equal or above 0.40 in absolute magnitude. This model considered that the antisocial, borderline, histrionic, narcissistic, and obsessive-compulsive PDs were associated with the first latent variable, that the avoidant and dependent PDs were associated with the second latent variable, that the paranoid and schizotypal PDs were associated with the third latent variable, and that the schizoid PD was associated with the fourth and last latent variable. Secondary loadings of the antisocial and histrionic PDs on the fourth latent variable, and of the borderline PD on the second latent variable, were also taken into account. Finally, the four latent variables were allowed to covary.

Structural equivalence was associated with good or acceptable goodness-of-fit indices, χ^2^(52) = 182.48, *p* < 0.001, χ^2^/df = 3.51, TLI = 0.87, CFI = 0.93, RMSEA = 0.05. Requiring the model to also reach metric equivalence induced a significant decrease of the goodness-of-fit indices, Δχ^2^(9) = 61.24, *p* < 0.001 [χ^2^(61) = 243.73.48, *p* < 0.001, χ^2^/df = 4.00, TLI = 0.85, CFI = 0.90, RMSEA = 0.05], but this decrease was of modest amplitude. Finally, adding the constraint of also reaching scalar equivalence had a major negative impact on the goodness-of-fit indices, Δχ^2^(10) = 476.32, *p* < 0.001, which were very poor, χ^2^(71) = 720.05, *p* < 0.001, χ^2^/df = 10.14, TLI = 0.54, CFI = 0.64, RMSEA = 0.10. The Mooré version of the IPDE seems to reach structural and metric equivalence across regions, but certainly not scalar equivalence. For this reason, the small differences, with higher scores for the schizotypal and histrionic and lower scores for the borderline and narcissistic PD scales in Ouagadougou (η^2^ ≥ 0.01), the medium differences, with higher scores for the antisocial and lower scores for the dependent PD scales in Ouagadougou (η^2^ ≥ 0.06), and the large difference, with higher scores for the schizoid PD in Ouagadougou (η^2^ ≥ 0.14), cannot be interpreted, as the origin of these scales varies across regions.

## Discussion

Demographic variables only had a small impact on normal and abnormal personality traits. The structure underlying normal personality was unstable across regions and languages, illustrating the fact that translating a complex psychological inventory into a native African language is a very difficult task, even with the help of highly qualified professionals. The structure underlying abnormal personality was stable across regions, with scales even reaching metric equivalence. As scalar equivalence was not reached, mean differences cannot be interpreted. This lack of scalar equivalence might be due to a method bias (slightly different interviews in Ouagadougou and Soumiaga) or to a real cultural difference between the rural and the urban region. Indeed, these mean level differences could be due to an exaggerated expression of abnormal traits valued in the two cultural settings studied. Our results suggest that studies using a different methodology should be conducted to understand what is considered, in different cultures, as deviating from the expectations of the individual’s culture, and as a significant impairment in self and interpersonal functioning, as defined by the DSM-5.

Internal consistencies, correlations with age, gender differences were globally low, compared to what is usually observed in Western cultures (McCrae et al., 2005), but similar to what was observed in a previous study conducted in Burkina Faso (Rossier et al., 2005). For PDs, these consistencies are low partly due to the heterogeneity of the symptomatology and comorbidity of these disorders and are similar to the consistencies found in other studies using the same screening questionnaire (Frances, 1982; Verardi et al., 2008). For most domains, in most sub-samples, no difference between women and men was observed. When a difference was observed the associated effect size was always small. Gender differences thus seem especially small in Burkina Faso in this context. In a previous article we explained that these low internal consistencies, correlations with age, and gender differences could be due to a range restriction associated with the fact that behaviors might be determined more by the social and cultural context in a collectivistic culture (Rossier et al., 2005). Cultural values, but also familiarity with personality questionnaires or surveys, might have an impact on the way people are responding. The rural sample in this study was living quite far away from the capital of the country, and was certainly less used to being interviewed than the sample from Ouagadougou. This might partly explain the difference in the response pattern of the rural sample, characterized by less heterogeneity and an even more pronounced range restriction that the restriction usually observed when using French questionnaires in Burkina Faso (Rossier et al., 2008).

The structure underlying the Mooré version of the NEO-PI-R administered through interview was clearly different to the structure postulated by the five-factor model of personality. In Mooré the interpersonal dimensions – extraversion, agreeableness, and openness – were highly correlated (*r* ≥ 0.46). The correlation was especially high between extraversion and openness (*r* = 0.71). Interestingly the difference in terms of structure across languages was as important as that observed across regions. This is certainly partly due to translation problems. It should be noted that Mooré is mainly a oral language which slightly changes from one region to the other. For this reason, we had to adapt our Mooré translation for the field trials in Ouagadougou and Soumiaga. These adaptations may explain the low replicability across regions. This results contrast strikingly with the fact that the five-factor model replicates in Mossi samples when using a French-version of the NEO-PI-R (Rossier et al., 2005). This could confirm a translation bias but also might be attributed to slightly different personality markers within the Mooré language, suggesting that a combined emic and etic approach might be appropriate to study personality this type of cultural settings (Zecca et al., in press). The structure underlying PDs using the French-version of the screening questionnaire and the structure underlying the Mooré version were relatively different. This poor replicability across languages might be attributed to the small group of participants who answered the French questionnaires, to a language bias (translation problems), or to a method bias (questionnaires vs. interviews). Indeed, answering a questionnaire or being interviewed are two very different situations. Social norms might have a different impact in these two situations. The structures obtained in Ouagadougou and in Soumiaga using the Mooré interview were very similar considering that PDs are supposed to be context-dependent. However, this structure was quite different from the structure of Livesley et al. (1998) or Rigozzi et al. (2009).

Across regions, PD scales did not reach scalar equivalence, but reached metric equivalence. This level of equivalence might be surprising for traits supposed to be partly culturally determined. As scalar equivalence was not reached, mean differences should not be interpreted. Nevertheless, these differences are unusually large, considering that the interviews were roughly the same in both regions. Considering only the differences associated with a medium or a large effect size, individuals in a more Westernized and collectivist culture scored higher on the antisocial and schizoid PD traits, whereas individuals in a more traditional society scored higher on the dependent PD trait. Although this reveals not a true difference, the hypotheses of Paris (1998) were not supported. On the contrary, the idea that these disorders represent an overexpression of valued traits seems more plausible, as already suggested by Ruth Benedict and Margaret Mead, and as observed for women who overexpress dependent, histrionic and borderline PD traits (Crews et al., 2007; Kirmayer, 2007). To formally compare these two sets of competing hypotheses, a multicentric research should be conducted. The difficulty in such as study would be to define and specify the relationships between culture-related descriptors and PD traits. Concerning the differences observed in the present study, they might also be due to an undefined bias, such as a method bias linked to the education level and background of the interviewers. Finally, the lack of scalar equivalence confirms that standardized screening questionnaires and standardized interviews have major limitations when they are used in different cultural groups. An unidentified bias might just make scores unreliable (Van Ommeren et al., 2000).

This study has several important weaknesses which have already been discussed. Nevertheless, for future research various alterations of the research protocols might increase the reliability of the results. In this study a self-assessment inventory was administered as an interview. The use of an interview will certainly be more appropriate to assess PDs and normal personality, using for example the structured interview for the five-factor model of personality (Trull and Widiger, 1997). Moreover, considering that scalar invariance is difficult to reach when conducting a study in two very different cultural settings, an anthropological method taking more specifically into account the social context of PDs and broader social processes might lead to interesting results. This type of approach might also allow identifying potential culture-specific PD symptoms. An anthropological approach or a combined emic and etic approach could allow studying the culturally shared representations about human nature’s personality, such as Sow’s (1977, 1978) African personality model which includes ancestors. Anthropologists are usually interested in behavioral patterns shared by a group of persons, and these patterns can be compared to a more psychological analysis of people’s behaviors in specific situations.

One interesting question that remains open is to know whether the DSM-5 will make cross-cultural comparison easier. Diminishing the number of PD types will make them more distinct and easier to identify. We can also hope that DSM-5’s clusters of symptoms will be more homogeneous. In the coming years, it will be important to study the cross-cultural replicability of PD types and of the five domains and traits proposed by the DSM-5. Characterizing a person using PD traits and levels of impairment in self and interpersonal functioning implies defining cut-off values or measuring distances. Defining these values is very difficult, because they may partly depend on cultural, social, and personal expectations. Thus the distinction between normality and abnormality can be culture-specific and different in Africa compared to Europe (Margetts, 1968). This difference might be due simply to the fact that PD traits do not reach scalar invariance or might be attributed to actual differences. In this case, normality might be relative and has to be defined specifically for each culture (Benedict, 1934).

## Conflict of Interest Statement

The authors declare that the research was conducted in the absence of any commercial or financial relationships that could be construed as a potential conflict of interest.
